# Histological subtype of lung cancer in relation to socio-economic deprivation in South East England

**DOI:** 10.1186/1471-2407-8-139

**Published:** 2008-05-19

**Authors:** Victoria A Bennett, Elizabeth A Davies, Ruth H Jack, Vivian Mak, Henrik Møller

**Affiliations:** 1 King's College London, Thames Cancer Registry, 1st Floor Capital House, 42 Weston Street, London, SE1 3QD, UK

## Abstract

**Background:**

Previous studies have found differences in the histological subtypes of lung cancers affecting males and females. Our objective was to investigate trends in the incidence of histological subtypes of lung cancer in males and females in relation to socio-economic deprivation in South East England.

**Methods:**

Data on 48,031 males and 30,454 females diagnosed with lung cancer between 1995 and 2004 were extracted from the Thames Cancer Registry database. Age-standardised incidence rates for histological subtypes were calculated for each year, using the European standard population. Using the Income Domain of the Multiple Index of Deprivation 2004, patients diagnosed between 2000 and 2004 were classified into quintiles of socio-economic deprivation based on their postcode of residence. Age-standardised rates for each histological subtype were then calculated for each deprivation quintile. A Poisson regression model was fitted to the data for males and females separately to test the hypothesis that the relationship between socio-economic deprivation and adenocarcinoma was less strong than for other subtypes.

**Results:**

In males all specific histological subtypes except adenocarcinoma declined in incidence. Squamous cell carcinoma remained the most common specific subtype and large cell carcinoma the least common. In females squamous cell carcinoma was initially most common, but its incidence declined slightly and that for adenocarcinoma increased. In both sexes the overall age-standardised incidence rate of lung cancer increased with increasing deprivation. However, these trends were less strong for adenocarcinoma than for the other subtypes in both males (p < 0.001) and females (p = 0.003).

**Conclusion:**

The temporal trends and distribution of histological subtypes of lung cancer in males and females are similar to that reported from other western populations. In both males and females, adenocarcinoma was less strongly related to deprivation than other subtypes. This may be because its development is less strongly linked to individual smoking history.

## Background

Lung cancer is the most common cause of cancer death in both males and females in the United Kingdom [1]. The age-standardised incidence rate of lung cancer has been decreasing in males since approximately 1980, but in females it has continued to increase, although some analyses have shown that this may now be reaching a plateau [1]. Data from 2001 to 2003 for the United Kingdom showed a lung cancer incidence of 64.5 per 100,000 population in males and a mortality rate of 57.4. In females the incidence was 34.8 per 100,000 population and the mortality rate was 29.6 [2]. The main cause of lung cancer is smoking [3].

Differences have been observed in the histological subtype of lung cancers affecting males and females and in their distribution over time. In particular, the proportion of adenocarcinoma tends to be higher in females [4]. Previous studies have shown changes over time in the distribution of histological types of lung cancer [4]. In Scotland, the proportion of adenocarcinoma in females increased between 1975 and 1997 [5]. Adenocarcinoma is less strongly associated with smoking than the other types of lung cancer [6]. Small cell carcinomas are most closely linked to smoking, followed by squamous cell carcinoma, and large cell carcinoma. It has been suggested that an increase in adenocarcinoma in smokers may be due to increased use of low tar cigarettes [5]. These may be associated with deeper inhalation, allowing carcinogens to reach the more peripheral parts of the lungs, where adenocarcinoma tend to occur [7-9].

There is a strong relationship between socio-economic deprivation and the incidence of lung cancer, with the most deprived areas having the highest incidence [10-13]. In the United Kingdom one study has found that this relationship is stronger in females than in males [14], while another has found it is the same in both sexes [15]. It is possible that differences exist in the incidence of histological subtypes across socioeconomic groups, which could shed light on their aetiology.

This study investigates recent trends in the incidence of the main histological subtypes of lung cancer, and their relation to socio-economic deprivation in South East England. Its objectives were

1) To describe and compare trends in the age-standardised rates of different histological subtypes in males and females between 1995 and 2004.

2) To compare socio-economic gradients in the incidence of lung cancer in males and females between 2000 and 2004.

3) To compare socio-economic gradients in the incidence of different histological subtypes in males and females, between 2000 and 2004.

## Methods

In the United Kingdom cancer registries record the occurrence of cancer in their resident populations. During the study period, the Thames Cancer Registry covered a population of 14 million people living in the area of South East England covering Essex, Hertfordshire, London, Kent, Surrey and Sussex. In this area, cancer registration is initiated by clinical and pathological information received from hospitals and by information about deaths provided by the National Health Service Central Register through the Office for National Statistics. Trained data collection officers collect further information on demographic details, disease stage and treatment from the medical records of individual patients. Data are continuously added to a central database and quality assured.

Data on 48,031 males and 30,454 females diagnosed with primary lung cancer in the period 1995 to 2004 were extracted from the Thames Cancer Registry. Age-standardised incidence rates were computed for each year of incidence, using the European standard population. Using the Income Domain of the 2004 Indices of Deprivation [16], patients diagnosed between 2000 and 2004 were classified based on their postcode of residence into quintiles of deprivation. This index is commonly used in England for national and local analysis and we chose to use the income domain because this is closely related to smoking rates and therefore to lung cancer rates. We chose to concentrate on the years 2000 to 2004 so that we could use the latest available deprivation indices. We also did not wish to make the assumption that these areas had the same level of deprivation in the earlier years 1995 to 1999. Age-standardised rates were computed for each histological subtype by deprivation category.

A Poisson regression model was fitted to the data separately for males and females with the following covariates: age (categorical), deprivation (one linear term), histological type (adenocarcinoma; other). The hypothesis that the relationship to socio-economic deprivation was less strong for adenocarcinoma than for the other groups was tested by including the deprivation * histology interaction term in the regression model.

Cancer registries in the United Kingdom carry out cancer surveillance using the data they collect under Section 60 of the Health and Social Care Act 2002. The study used an anonymised dataset and separate ethical approval was not required.

## Results

**Figure 1:**
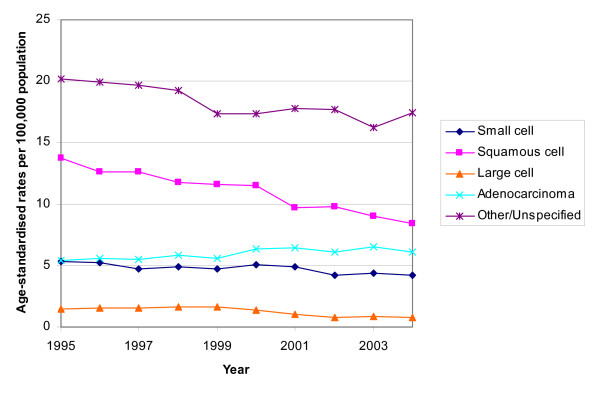
Trends in age-standardised rates of lung cancer by histology in males, South East England, 1995 – 2004.

**Figure 2:**
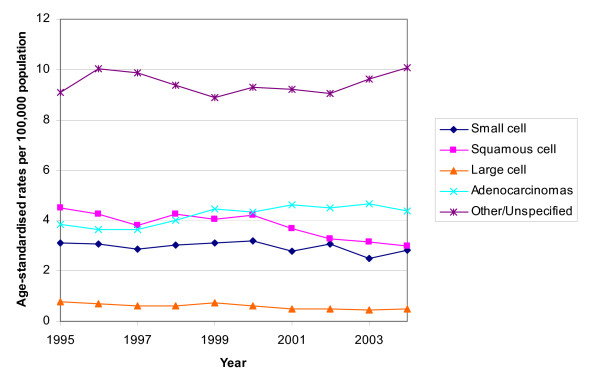
Trends in the age-standardised rates of lung cancer by histology in females, South East England, 1995 – 2004.

In males the age-standardised rates decreased over the period 1995 to 2004 for all histological subtypes, except for adenocarcinoma. Squamous cell carcinoma remained the most common specific type and large cell carcinoma the least common (Figure 1). In females the most common subtype was initially squamous cell carcinoma but its incidence declined slightly and that for adenocarcinoma increased (Figure 2). The incidence of small cell, large cell and "other and unspecified" carcinomas remained relatively constant. There was a relatively high proportion of unspecified cancers.

**Figure 3:**
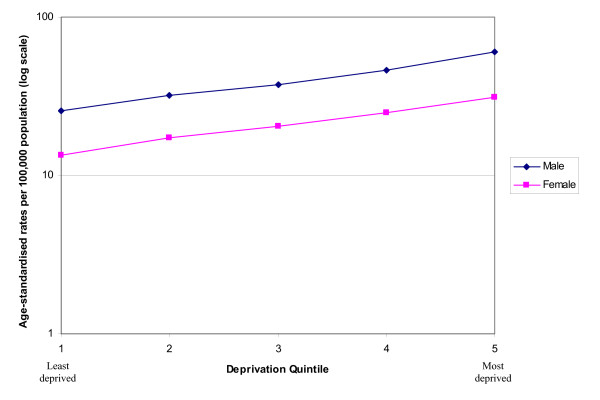
Age-standardised rates of socio-economic lung cancer by deprivation, South East England, 2000 – 2004.

The overall age-standardised incidence rate of lung cancer increased with increasing deprivation (Figure 3), and the same magnitude of change was seen in males and females. In males, the incidence increased from 25.5 per 100,000 in the least deprived quintile to 60.3 per 100,000 in the most deprived quintile. In females the incidence increased from 13.4 per 100,000 in the least deprived quintile to 31.1 per 100,000 in the most deprived quintile.

**Figure 4:**
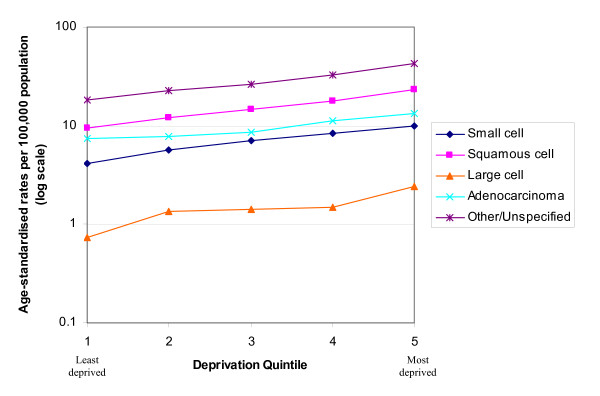
Age-standardised rate of lung cancer by socio-economic deprivation in males, South East England, 2000–2004.

**Figure 5:**
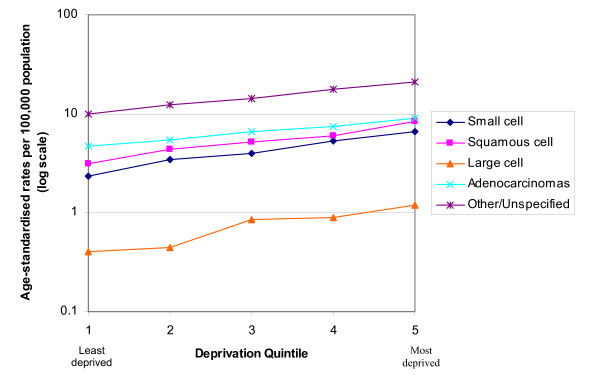
Age-standardised rate of lung cancer by socio-economic deprivation in females, South East England, 2000 – 2004.

In both males and females similar rates of increase with increasing deprivation were seen in the incidence of all the histological subtypes except for adenocarcinoma (Figures 4 and 5). The incidence of adenocarcinomas was less strongly linked to deprivation than for the other subtypes in both males (p < 0.001) and females (p = 0.003). Table 1 shows the exact data for the age-standardised rates which are plotted in Figures 4 and 5.

**Table 1:** Age-standardised rates per 100,000 for lung cancer histological subtypes, males and females by deprivation quintile

	Males					Females				
		
Deprivation Quintile	Small cell	Squamous cell	Large cell	Adeno- carcinoma	Other/Unspecified	Small cell	Squamous cell	Large cell	Adeno- carcinoma	Other/Unspecified
Most affluent 1	4.12	9.52	0.74	7.35	18.31	2.36	3.14	0.41	4.68	9.90
2	5.63	11.98	1.35	7.71	22.90	3.46	4.42	0.44	5.47	12.18
3	7.05	14.71	1.43	8.61	26.45	3.97	5.15	0.84	6.56	14.08
4	8.32	17.87	1.48	11.10	32.54	5.29	5.97	0.90	7.48	17.58
Most deprived 5	9.89	22.99	2.44	13.44	42.77	6.52	8.38	1.19	8.97	21.03

## Discussion

This study using cancer registration data for South East England found that the incidence of squamous cell carcinoma fell and adenocarcinoma rose between 1995 and 2004. The same rate of increase in lung cancer incidence with increasing deprivation was seen in males and females. However, adenocarcinoma was less strongly associated with deprivation than the other histological subtypes.

The accuracy of the data used for this study is dependent on that recorded within hospitals and the completeness of its extraction by cancer registry data collection officers. The analysis of histological subtypes is also limited by the large proportion of unspecified lung cancers. However, the age-standardised rate of unspecified lung cancers from 2000–2004 has decreased in males and remained relatively constant in females. This is the same general trend seen in the other cancer subtypes which suggests that the unspecified lung cancers have not obscured any trends. Also, the study size is large and uses the most detailed information available for this geographical region and time period. Nonetheless this is a weakness of the study, particularly if histological type is missing more often from some deprivation quintiles than from others. This kind of bias would obscure the actual trends. For this reason these trends should be explored in datasets from other countries that are more complete.

The finding of a change over time in the pattern of histological subtypes of lung cancer is consistent with previously reported findings [4,5]. The increase in adenocarcinoma and decrease in squamous cell carcinoma has previously been observed and attributed to an increase in low tar cigarette use [8,17]. It has been suggested that people who smoke low tar cigarettes tend to inhale more deeply, and that this allows carcinogens to reach more peripheral parts of the lungs where adenocarcinoma occur [9]. Since smoking is the main cause of lung cancer, smoking cessation has also played a role in the changing trends. Adenocarcinoma is the subtype that is least strongly associated with smoking, therefore smoking cessation will have less effect on its incidence than the other histological subtypes. Some changes in histology may also be explained by improvements in diagnosis. For example, new biopsy techniques have improved access to the periphery of the lung and therefore increased the diagnosis of adenocarcinoma. Other authors have concluded that the increase in adenocarcinoma is not explained by these changes [18].

This study found that lung cancer incidence had the same magnitude of association with socio-economic deprivation in both males and females. One previous study in England has shown that lung cancer had a greater magnitude of association with socio-economic deprivation in females, than in males. The incidence in females was three times higher in the most deprived group than in the most affluent, whereas in males the incidence was two and a half times greater in the most deprived [14]. Another study in Scotland found a three-fold increase in incidence in both men and women in the most deprived groups compared to the most affluent [15]. However, both studies used the Carstairs deprivation index, and each reported the findings in a slightly different way which makes it difficult to compare the results directly with our own. Smoking is the main cause of lung cancer, and in England smoking prevalence increases with socio-economic deprivation [2]. Although a slightly lower percentage of females than males smoke in each socio-economic group, the same gradient of increased smoking with increased deprivation is seen [2]. As smoking is the main cause of lung cancer this would help to explain why deprivation had the same effect on both males and females. Some previous research has suggested that fruit and vegetable intake may be important in determining lung cancer risk. A study in the Netherlands suggested an association of diet with lung cancer incidence. The authors found that increased consumption of ß carotene, vitamin C, vegetables, and fruit was associated with a decreased risk of lung cancer, but found no apparent relationship with vitamin E [19]. This hypothesis remains controversial.

The major point of interest when looking at histological subtype and deprivation is that adenocarcinoma is less strongly linked to socio-economic deprivation. Further research investigating this relationship in other populations would be worthwhile. Increased levels of smoking are thought to explain the increased lung cancer incidence with increased socio-economic deprivation. The weaker association between adenocarcinoma incidence and deprivation may be because this subtype is less strongly linked to individual smoking history.

## Conclusion

The distribution of lung cancer histological subtypes in males and females is similar to that reported in other western populations. In this study adenocarcinoma was less strongly related to deprivation than other subtypes, possibly because its development is less strongly linked to individual smoking history.

## Competing interests

The authors declare that they have no competing interests.

## Authors' contributions

VB conceived of the study, helped design it, analysed the data and wrote the first draft of the paper. ED helped interpret the findings and revised subsequent drafts of the paper. RJ helped to design the study, analyse the data, interpret the findings and commented on the paper. VM helped analyse the data, interpret the findings and commented on the paper. HM helped to design the study, interpret the findings and write the paper.

## Pre-publication history

The pre-publication history for this paper can be accessed here:


